# On-Sight and Red-Point Climbing: Changes in Performance and Route-Finding Ability in Male Advanced Climbers

**DOI:** 10.3389/fpsyg.2020.00902

**Published:** 2020-05-28

**Authors:** Eloisa Limonta, Maurizio Fanchini, Susanna Rampichini, Emiliano Cé, Stefano Longo, Giuseppe Coratella, Fabio Esposito

**Affiliations:** ^1^Department of Biomedical Sciences for Health, University of Milan, Milan, Italy; ^2^IRCCS Galeazzi Orthopedic Institute, Milan, Italy; ^3^Department of Neurosciences, Biomedicine and Movement Sciences, University of Verona, Verona, Italy

**Keywords:** sport climbing, lead climbing, bouldering, route preview, movement sequence recall, climbing performance, climbing style

## Abstract

**Aim:**

In lead climbing, the ascent of the route can be defined as on-sight or red-point. On-sight is the more challenging style since it demands greater physiological and psychological commitment. The differences between the two modes in advanced climbers have not been studied much. Two essential skills needed to optimize performance, in both on-sight and in red-point climbing, are route interpretation (RI) ability and movements sequence recall. Therefore, this study aimed to compare performance between on-sight and red-point ascent in advanced climbers and evaluate how a climber’s RI ability and movement sequences recall might change before and after on-sight and red-point climbing.

**Methods:**

Eighteen advanced male climbers (age 29.2 ± 4.7 years, body mass 67.8 ± 3.6 kg, stature 175.2 ± 2.4 cm, best red-point and on-sight grades 7b+/8a and 7a+/7b+, respectively) were video-recorded during the route ascent in on-sight and red-point modes to evaluate performance and to measure static and dynamic action times. RI ability and movement sequence recall were assessed before and after each climb. Level of anxiety was evaluated via a self-report questionnaire. Heart rate (*f*_H_), lactate concentration, ([La^–^]), and rating of perceived exertion (RPE) were detected during and after each climb.

**Results:**

Compared to on-sight, an improvement in performance was observed in a red-point climb: the ascent was faster (148.7 ± 13.6 s and 179.5 ± 12.5 s, respectively, *P* < 0.05), smoother (significant reduction in exploratory moves and in stops times, *P* < 0.05), less demanding physiologically (lower *f*_H__peak_ and [La^–^]_peak_, *P* < 0.05), and psychologically (lower RPE, cognitive and somatic anxiety and higher self-confidence, *P* < 0.05). The RI ability was improved in red-point versus on-sight and, in the same mode, between pre and post ascent.

**Conclusion:**

Red-point climbing was found to be less demanding than on-sight, both physiologically and psychologically, under the conditions investigated by this study. Our findings suggest that RI is a trainable skill and underscore the importance of including specific techniques in training programs designed to improve interaction between perceptual, psychological, and physiological factors.

## Introduction

Sport climbing is an emergent discipline that will be included for the first time in the 2020 Olympic Games official program in Tokyo. The fast growth of climbing as a competitive sport has attracted research interest as well. This multidimensional activity differentially incorporates physiological and psychological skills (Morrison and Schöffl, 2007; Hodgson et al., 2009; Draper et al., 2011) in three distinct specialties: lead, boulder, and speed. In lead climbing, the climber’s goal is to move vertically until the end of the itinerary (pitch or route) outlined on an artificial wall with handholds and footholds. The ascent can be performed in one of two styles depending on the safety procedures: lead or top-rope. In lead climbing, the climber secures his ascent at the belays prepositioned throughout the wall, using a safety rope fitted to a harness (Orth et al., 2016). In case of a mistake or exhaustion, the climber will fall below the last anchor point used. In top-rope climbing, the rope is passed through an anchor at the top of the route prior to the ascent (Orth et al., 2016). In case of error or exhaustion, the climber will not fall but will remain hanging by the rope. Moreover, as regards knowledge of the route, the ascent can be defined as on-sight or red-point mode. The climb is on-sight when the pitch is lead first time without a single fall, without any previous practice and without the climber having any useful information about the characteristics of the route (Sanchez et al., 2019). The climber is allowed to visually inspect the on-sight ascent from the ground up, but with no prior pointers from an outside source. Any subsequent attempt to the climb is referred to as red-point.

For climbers, the purest and more demanding style of ascent is an on-sight lead climbing (Draper et al., 2008) since it involves greater physiological and psychological commitment due to the fear of falling and lack of knowledge of the route characteristics (Aras and Akalan, 2014). Being the most challenging approach, the on-sight lead climbing is the standard style used during final rounds of lead competitions, according to the International Federation of Sport Climbing rules (International Federation of Sport Climbing, 2020).

Sport climbing has become the recent subject of scientific studies, with most having focused on its physiological aspects and a few others having assessed the level of psychophysiological stress associated with different climbing styles (lead and top-rope, on-sight, and red-point) (Draper et al., 2008, 2011; Hodgson et al., 2009; Fryer et al., 2013; Aras and Akalan, 2014).

Overall, comparison between leading and top-rope climbing has shown that novice and intermediate climbers find leading climbing more stressful both physiologically and psychologically than top roping (Hardy and Hutchinson, 2007; Draper et al., 2008; Hodgson et al., 2009; Aras and Akalan, 2014), whereas advanced and elite climbers don’t seem to experience significantly greater anxiety in lead than in top-rope climbing (Fryer et al., 2013).

To our knowledge, only one study to date has analyzed the differences between on-sight and red-point climbing (Draper et al., 2008) and found that climb times, lactate concentrations, and self-reported pre-climb somatic and cognitive anxiety are higher in on-sight mode. Lack of information about and experience of the pitch elicit greater anxiety about falling and impair route interpretation (RI), problem solving ability, and movement sequence recall (Watts, 2004; Giles et al., 2006).

In on-sight lead climbing, RI strategies of the climber before the ascent and problem-solving ability during the ascent are the essential skills needed to optimize the performance (Boschker et al., 2002; Sanchez et al., 2012; Ferrand et al., 2006). For this reason, during competition and training, climbers visually inspect the route from the ground (route preview), in order to understand and visualize the optimal sequence of movements they will need to climb it. Route preview errors can be considered one of the major reasons for falling during climbing (Boschker et al., 2002; Sanchez et al., 2012). In red-point lead climbing, the ability to memorize (i.e., retain the information acquired during the on-sight ascent) is also a crucial skill (Sanchez et al., 2012). Therefore, exploratory behavior, which is influenced by past experiences and motor, perceptive, and mnemonic skill, is a potential indicator of learning and performance. Though route preview and recall ability are fundamental skills, whether and how they are trainable has not yet been determined. Hence, this study had two aims: to compare the performance during on-sight and red-point leading climbing in advanced climbers, on a route matching their best on-sight skill; and to evaluate change from before and after on-sight vs. red-point lead climbing performances related to RI and movement sequences recall ability in advanced climbers.

## Materials and Methods

### Subjects

Eighteen male climbers participated in this study. All were climbing instructors, with a coaching experience of at least 4 years. Two were excluded from the study because of inadequate performance in the trials, so the final sample was 16 participants. As described in Draper et al. (2011), they were classified as advanced on the basis of their self-reported best red-point and on-sight rates, for the last year, 7b+/8a and 7a+/7b+,respectively, according to the French Rating Scale of Difficulty. The average age was 29.2 ± 4.7 years (range: 24–35 years) and the average height and body mass were 175.2 ± 2.4 cm (range: 172–179 cm) and 67.8 ± 3.6 kg (range: 64–73 kg), respectively. Their climbing experience was 9.2 ± 3.8 years and training frequency was 11.2 ± 3.2 h/week.

At the time of the study, all were clinically healthy with no musculoskeletal disorders. After receiving a full explanation of the experimental procedures and aims of the study, they gave their written, informed consent to participate. The study was approved by the University of Milan ethical committee and performed in accordance with the principles of the 1975 Declaration of Helsinki.

### Experimental Protocol

Two experimental sessions were conducted 1 week apart, in an indoor climbing gym. For each session, the participants were asked to refrain from caffeine or other similar beverages for at least 4 h prior to testing and to refrain from any form of strenuous physical exercise in the previous 48 h. All sessions were completed as lead climbs.

In the first session, the participants attempted the on-sight condition. The difficulty of the pitch was close to their best on-sight level (7a+/7b+) but they were unaware of the degree. A professional certified route setter was used on an artificial indoor climbing wall in such a way that technical and physical difficulties were distributed along the entire route. All the routes were similar in average slope (10 ± 2° overhanging), overall length (17 ± 2 m of maximal height), and number of handholds (38 ± 2).

In the second session, the participants climbed the same route, now in red-point mode.

Before and after the two ascents (on-sight and red-point), the participants were assessed for their RI and movement sequence recall abilities and evaluated for their state of anxiety via a self-report questionnaire. The climbing performance, moreover, was assessed by ascent time, rate of perceived exertion (RPE), and physiological parameters (heart rate, *f*_H_; lactate concentration, [La^–^]).

### Experimental Procedures

**FIGURE 1:**
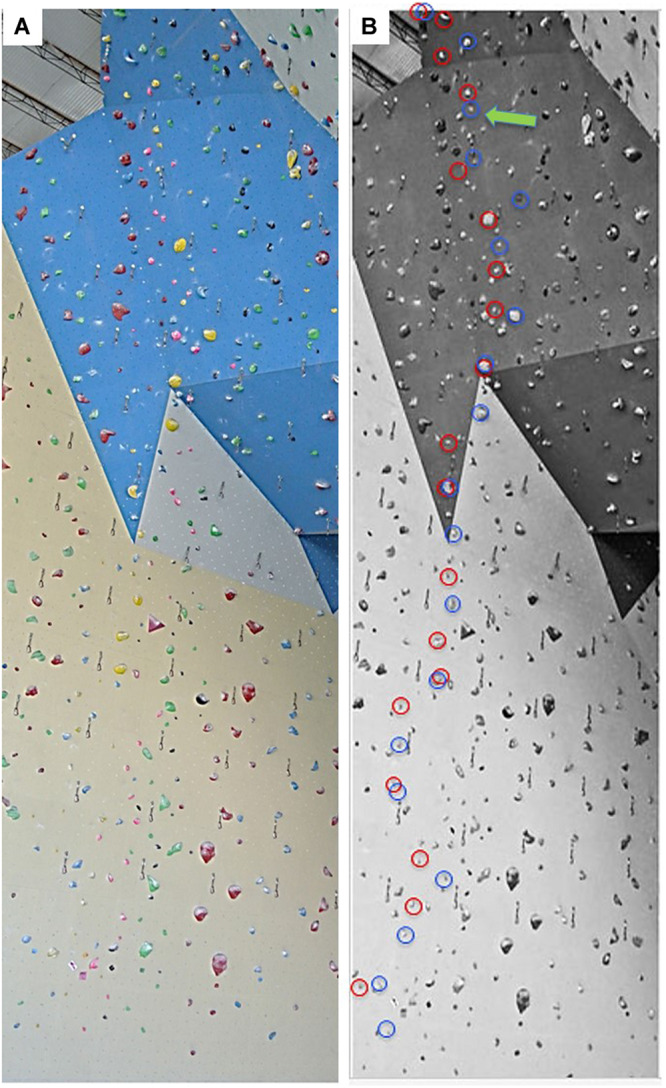
Artificial indoor climbing wall with one of the routes used for the test (yellow, 7b) **(A)**. The black and white image **(B)** is the same image as the one shown to the climbers before and after the on-sight and the red-point ascent. The handholds that climbers planned to grasp are circled (red = left hand, blue = right hand). The green arrow indicates the spot where a climber expected to fall.

In the first session, at rest, *f*_H_ was monitored by electrocardiography (Mod. Delta 1 PLUS, Remco Italia Cardioline, Italy) and whole blood lactate concentration was measured on an enzymatic amperometric system (LabTrend, BST Bio Sensor Technology, Berlin, Germany). The lactameter was calibrated and checked against standard solutions before each trial to ensure consistent data. The blood samples (20 μL) were collected from the little finger of each climber to minimize the impact on grip during the climb. The RPE was measured on a 6–20 Borg scale (Borg, 1982). After a semi-standardized warm-up (which included jogging, dynamic mobility exercises, and a lower grade practice climb), the participants were allowed 6 min to preview a route. Although set up on an artificial wall, together with others, the pitch was easily recognizable by the color of the holds. After previewing the route, the participant sat in front of a computer screen that displayed a black and white image of the wall (Figure 1). To test his route identification (RI_pre on–sight_) ability, the participant was asked to identify the holds (handholds and footholds) of the pitch, to simulate a sequence of movements, and how he would grasp each hold. He was also asked to make an estimate about his ability to either complete the route successfully or where he might fail. The participants then completed the Competitive State Anxiety Inventory 2 (CSAI-2). The CSAI-2 is a sports-specific anxiety inventory that comprises three 9-item subscales that measure cognitive anxiety (mental component of anxiety caused by negative expectations about success or negative self-evaluation), somatic anxiety (associated with the physiological or affective component of anxiety), and self-confidence (i.e., self-efficacy perception) (Martens et al., 1990; Cox et al., 2003). Each item is scored on a four-point scale from 1 (not at all) to 4 (very much so). The total scores range for each subscale is from 9 to 36, with 9 indicating low anxiety (high confidence) and 36 indicating high anxiety (low confidence).

Then, on lead, each participant climbed the route as an on-sight. The climbers were given no specific ascent instructions, except to self-pace and climb fluently. If a fall occurred prior to finishing two-thirds of the route or in the last third of the route, the climber abseiled to the ground and the trial data were either excluded or included, respectively, from the final analysis. Accordingly, the data from two of the 18 participants were subsequently excluded from the analysis.

The ascents were video-recorded with a digital video camera and timed. During the climb, *f*_H_ was monitored continuously by electrocardiography. Immediately after finishing the climb, the RPE was assessed, the blood lactate concentration was measured at minutes 1, 3, and 5, and the route identification test (RI _post on–sight_) was re-administered.

In the second session, after the *f*_H_ and [La^–^] measurement at baseline and RPE assessment, the participants performed the same warm-up as in the first session. Subsequently, each climber had a total of 60 min and a maximum of four attempts to ascent and study the route. At the end of this time, the RI_pre red–point_ and the CSAI-2 were re-administered. Finally, on lead, the climbers completed the red-point condition, and immediately post-climb retook the RI_post red–point_ test.

### Data Analysis and Statistic

Statistical analysis was performed using a statistical software package (SigmaStat for Windows, v3.11, Systat Software Inc., United States). A Kolgomorov–Smirnov test was applied to check for normal distribution of the data. A sample size of 14 participants was selected to ensure a statistical power higher than 0.80.

The ascent time in the two climbing modes was measured. The start was set when the climber took his feet off the ground and the end was set when he passed his safety rope in the chain at the top of route or where he fell. In the event of a fall, the climb time for the pitch common to the modes was entered in the data analysis. To evaluate the accuracy of a climber’s prevision of success, the highest number of the handholds grasped was compared against the estimated pre-climb number.

To evaluate the climbing performance, static and dynamic actions were counted (Flahaut and Loslever, 2000; Pijpers et al., 2005), as applied in sport climbing analysis by Sanchez et al. (2012). The coding of performance consisted of measuring the duration of movements during the climb, divided into: performatory (necessary for ascent, measured from the release of a handhold to the moment of contact with another handhold) or explorative (unnecessary for ascent but useful for choosing the next movement. The handholds and footholds were touched without being used as a support). The duration of stops was measured and divided into appropriate stop (partial resting points) or inappropriate stop (static phases due to indecisions or errors but not useful for resting). Two independent operators viewed the video-recordings, rated the participants’ performance, and compared it with the RI test.

Heart rate was monitored continuously from the basal condition to the end of the on-sight and the red-point climb. The basal (average over 1 min, in rest conditions, before warm-up), the peak during the climb (average over 10 s), and the average over the entire ascent time were calculated. [La^–^] level at baseline and peak after the ascent were measured.

To compare the RI test results and the video-recordings, for the on-sight and the red-point climbs we calculated the number of holds incorrectly identified on the black and white image shown on the computer screen, and the number of handholds grasped during the climb that differed from those previewed on computer screen image. The values are expressed as a percentage of the total number of holds taken during the route. From the RI test we also obtained the prevision of route success indicated by the participants before the red-point and the on-sight climbs, while from the video we verified the actual success.

Descriptive statistics [mean; standard deviation, (SD); standard error, (SE)] were used to describe the perceptive, psychological, and physiological variables, and the RI test results. Possible differences in perceptive, psychological, and physiological variables between the on-sight and the red-point climbs were checked using a one-way (modality) analysis of variance (ANOVA) for repeated measures. A two-way (time × modality) analysis of variance (ANOVA) for repeated measures was applied to determine differences in RI test results. The *post hoc* Bonferroni test was selected when necessary to locate the differences. Statistical significance was set at *P* < 0.05.

Pearson Correlation test was applied to identify associations between time to ascent, physiological parameters (*f*_H_, [La^–^]), and perceptual variables (RPE, cognitive anxiety, somatic anxiety, and self-confidence).

## Results

The ascent time was significantly shorter in red-point than in on-sight mode (148.7 ± 13.6 s and 179.5 ± 12.5 s, respectively, *P* < 0.05). Table 1 presents the performance variables (performatory and exploratory move time; appropriate and inappropriate stop times). Significantly fewer (*P* < 0.05) exploratory moves and appropriate/inappropriate stop times were observed for the red-point compared to the on-sight climb. Performatory move times were also shorter for the red-point climb, albeit not significantly.

**TABLE 1:** Total ascent time and duration of dynamic (performatory and exploratory moves) and static phases (appropriate and inappropriate stops time) in the on-sight and the red-point climb.

	On-sight lead climb	Red-point lead climb
*Total ascent time* (s)	179.5 ± 12.5	148.7 ± 13.6*
*Performatory moves* (s)	141.4 ± 11.0	128.8 ± 12.1
*Exploratory moves* (s)	15.4 ± 9.6	6.7 ± 4.2*
*Appropriate stops* (s)	42.2 ± 11.4	31.0 ± 8.9*
*Inappropriate stops* (s)	5.5 ± 2.3	1.2 ± 1.8*

Mean ± SD *P < 0.05 vs. on-sight.

Table 2 presents the physiological (*f*_H_, [La^–^]) and perceptual parameters (RPE, CSAI-2). There was no difference in baseline values between the two climbing modes. Significantly lower (*P* < 0.05) peak values of *f*_H_ and [La^–^] were measured after the red-point climb, whereas no difference in *f*_Hmean_ was observed between the two climbing modes.

**TABLE 2:** Physiological and perceptual parameters at baseline, during, and post on-sight and red-point lead climb.

		On-sight lead climb	Red-point lead climb

	Physiological parameters		
*f*_H_ (bpm)	*basal*	71 ± 4	67 ± 5
	*peak*	186 ± 7	175 ± 9*
	*average*	166 ± 8	160 ± 7
[La^–^](mmol/l)	*basal*	1.08 ± 0.40	1.02 ± 0.41
	*peak*	6.81 ± 1.78	5.06 ± 1.08*

	**Perceptive parameters**		

RPE	*basal*	8 ± 1	7.5 ± 1
	*post*	17 ± 1.5	15.5 ± 1*
CSAI-2 (pts)	*cognitive anxiety pre climb*	17.4 ± 3.2	12.0 ± 4.6*
	*somatic anxiety pre climb*	15.1 ± 4.2	11.3 ± 5.0*
	*self-confidence pre climb*	28.2 ± 3.4	30.8 ± 4.1*

f_*H*_, heart rate; [La^–^], blood lactate concentration; RPE, rate of perceived exertion; CSAI-2, competitive state anxiety inventory 2. Mean ± SD *P < 0.05 vs. on-sight.

Regarding perceptual parameters, the climbers declared a lower cognitive and somatic anxiety and a higher self-confidence before the red-point ascent. RPE was lower after the red-point climb.

Pearson correlation tests showed significant correlation between *f*_Hpeak_ and [La^–^] in both conditions (*r* = 0.638; *P* = 0.044 and *r* = 0.577; *P* = 0.048 in on-sight and red-point, respectively) and between [La^–^] and total ascent time (*r* = −0.338; *P* = 0.043 and *r* = −0.377; *P* = 0.039 in on-sight and red-point, respectively). Moreover, total ascent time in both conditions was correlated with cognitive (*r* = 0.541; *P* = 0.046 and *r* = 0.699; *P* = 0.024 in on-sight and red-point, respectively) and somatic anxiety (*r* = 0.854; *P* = 0.001 and *r* = 0.832; *P* = 0.002 in on-sight and red-point, respectively). No significant correlations were found among the other variables.

Figure 1 (panel A) displays the indoor climbing wall with one of the routes used for the trials (yellow holds, graded 7b according to the French Rating Scale of Difficulty). The black and white image on the right (Figure 1, panel B) is the same as that shown on the computer screen before and after the on-sight and the red-point climbs to assess RI ability. The participants were asked to identify all the holds on their route, indicating in red the handholds they planned to grasp with their left hand and in blue the ones they planned to grasp with their right hand. The point where a participant assumed he would fall was indicated by the green arrow.

**FIGURE 2:**
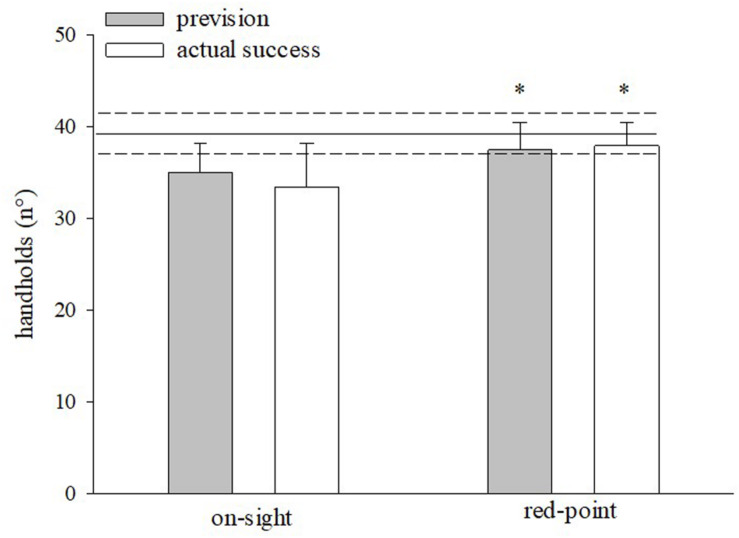
Number of the handholds grasped by the climber in the on-sight and the red-point ascent (before finishing the route or before falling) and the pre-climb prevision. Mean ± SD ^∗^*P* < 0.05 vs. on-sight.

Figure 2 compares the number of the handholds the participant grasped in the on-sight and the red-point climbs (before finishing the route or before falling) and the pre-climb prevision. The number of handholds grasped during the red-point climb was significantly higher (*P* < 0.05) compared to the on-sight, both for the pre-climb prediction and the actual climb. In both modes, the prevision was consistent with the effective results (i.e., with the real performance achieved by the climber).

**FIGURE 3:**
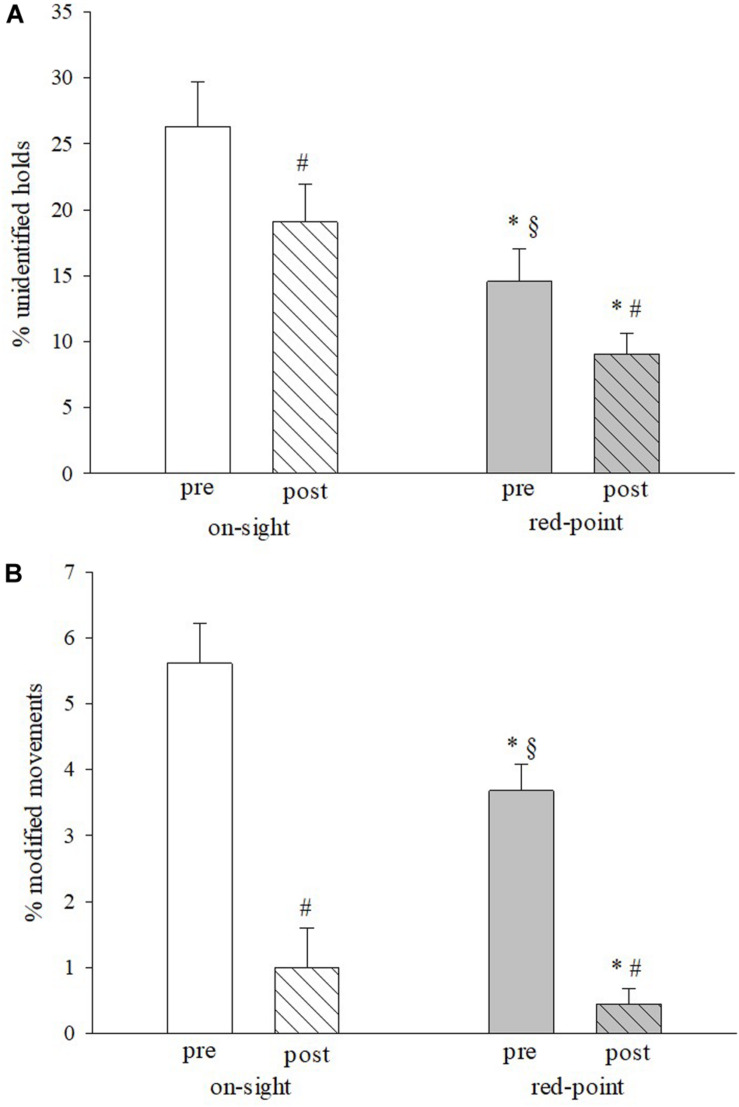
Percentage of incorrectly identified holds on the route **(A)** in the two test conditions and the percentage of the holds used differently during the climb from that stated on the RI test **(B)**. Mean ± SE ^∗^*P* < 0.05 vs. on-sight; ^#^*P* < 0.05 vs. pre; ^§^*P* < 0.05 vs. post on-sight.

Figure 3 presents the main results of the RI test. The percentage of incorrectly identified holds on the route (panel A) was significantly lower (*P* < 0.05) between pre and post red-point climbs compared to pre and post on-sight climbs. Fewer mistakes between pre and post ascent, in both styles, were observed (*P* < 0.05). The percentage of the holds used differently during the climb from the holds stated on the RI test was significantly reduced (*P* < 0.05) in the RI pre and post red-point compared to the pre and post on-sight climb. Significantly fewer mistakes (*P* < 0.05) in both climbing modes were made between the pre and the post trials. Between the RI _post on–sight_ and RI _pre red–point_ there was a lower percentage of unidentified holds and a higher percentage of modified moves (*P* < 0.05).

## Discussion

### Preliminary Considerations

To the best of our knowledge, this is the first study to compare physiological and perceptual parameters during on-sight and red-point climbing performance in advanced climbers on a maximum on-sight grade route. We chose a protocol that comprised different lead climbing styles as they might occur during indoor or outdoor climbing.

Climbers displayed an improvement in red-point performance, characterized by a smoother, faster, and more successful ascent. The physiological commitment and perceptual involvement showed a reduction in peak values, except for self-confidence in which there was an increase.

This is also the first study to evaluate how the route preview changes over time when climbing performance is repeated. Our results indicate that RI skills are trainable and that route preview is important not only before on-sight performance but also for optimization of red-point climbing.

### On-Sight and Red-Point Climbing Performance in Advanced Climbers

On-sight lead climbing is the more demanding style of ascent in sport climbing (Draper et al., 2008). When climbers attempt to push their limits beyond their current ability level during an on-sight attempt, they are unlikely to succeed without falling (Aras and Akalan, 2014). Next, they will begin to analyze the highly varied and individual movement sequences, focusing on the crux or personally most difficult sequential foot- and hand-hold arrangements. Once climbers deem their analysis of the route complete, they will attempt a red-point ascent, making use of the information and physical experience acquired during prior attempts. A climber’s ability to interpret a route, recall movement, and incorporate motor learning skills into the ascent is essential to reduce the number of attempts to reach the desired goal (Sanchez et al., 2019). A climber’s experience and level of performance strongly influence these abilities.

Only one study to date has compared the physiological and psychological aspects of an on-sight lead climb and a second lead climb (Draper et al., 2008). In their study, however, Draper and colleagues observed only novice, inexperienced climbers who climbed a route far below their skill level, which they were all able to finish in the on-sight attempt. Our study, however, involved highly experienced climbers, both athletes and instructors. Moreover, the route they climbed matched their best skills. Finally, between the first (on-sight) and the second (red-point) attempt, they had time to study and optimize the entire climb in all its parts, as normally done in sports climbing.

As expected, an improvement in performance between the on-sight and red-point climbs was observed, as demonstrated by the greater maximum height reached by climbers in the red-point than in the on-sight ascent (Figure 2) and by the significantly shorter ascent time (Table 1). With regard to the movements, there were significantly fewer exploratory moves and appropriate/inappropriate stop times in the red-point ascent. Moreover, performatory move time was also shorter in the red-point climb, albeit not significantly.

The move and stop times during climbing performance was compared by Sanchez et al. (2012) on pitches that differed in difficulty and were climbed in on-sight mode with or without route preview. They observed that while visual inspection does not influence how successfully a route is finished, it enhances movement fluency with faster speed, shorter stops, and less searching for foot and hand holds. These observations are in agreement with Dupuy and Ripoll (1989) who noted an improvement in expert climbers, with a shortening of pauses/inspection phases during a climb preceded by route preview, compared to one not previewed.

Our study adds interesting information about the influence of RI and the on-sight ascent on red-point climb. Experiential knowledge gained from preview and physical practice of the route enabled the climbers to considerably reduce the number of unnecessary moves and the static phases (appropriate and inappropriate stops). In addition, the climbers tended to perform the moves useful to the ascent faster, because they had practiced and optimized them. Both route preview and physical experience surely helped to make interpretation of the movement sequences faster and the execution smoother and more economic. Nevertheless, with this protocol, we were unable to define the influence of each aspect on the dynamic and static phases.

About the physiological parameters, peak *f*_H_ was significantly lower in the red-point ascent, whereas no difference in *f*_Hmean_ was observed between the two climbing modes. The lower peak *f*_H_ could be due to a reduced psychological involvement in the harder sections of the route, as observed in top-rope compared to lead climbing (Aras and Akalan, 2014). Moreover, the knowledge of the movement sequences makes climbing smoother and less expensive, limiting the *f*_H_ increase (Sanchez et al., 2012).

Peak blood lactate concentrations were lower in the red-point than in the on-sight climb. This could have been due to the optimization of moves and the reduction in stops times that imply a shorter duration of the upper limbs muscles in isometric, high-intensity contraction (Limonta et al., 2018). Isometric contractions are known to increase *f*_H_ and lactate concentration more than dynamic contractions, especially when the upper limb muscles are involved (Rowell et al., 1996; Astrand et al., 2003; Kuepper et al., 2009). As climbing speed increases, the isometric phase of upper limb muscles contraction shortens and this phenomenon may be attenuated (Limonta et al., 2018).

The perceptive parameters were in line with the physiological variables. The higher RPE at the end of the on-sight climb reflects the greater physical commitment (Pérez-Landaluce et al., 2002; Rodríguez-Marroyo et al., 2012) and anxiety it elicits (Morgan, 1994; Garcin et al., 2006). Anxiety is the chief psychological factor that affects athlete performance (Balagué, 2000; Craft et al., 2003; Woodman and Hardy, 2003; Aras and Akalan, 2014). Pijpers et al. (2005) observed significant differences in performance associated with high anxiety levels in novice climbers, but also élite climbers reported anxiety to be detrimental to successful performance because it induces rigid posture and jerky movements (Ferrand et al., 2006). The non-flowing movements lead to an increase in the physiological load which entails a greater energy expenditure and an inappropriate level of fatigue.

Cognitive and somatic anxiety were found to be higher and self-confidence lower in lead climbing compared to top-rope climbing (Aras and Akalan, 2014). Consistent with these findings, Draper et al. (2008) observed higher cognitive and somatic anxiety in on-sight lead climb, than in a second lead climb, with no differences in self-confidence.

Our data show less cognitive and somatic anxiety and greater self-confidence pre red-point climb compared to on-sight. The trends are consistent with previous studies, although the levels of cognitive and somatic anxiety seem to be lower on average and the self-confidence higher. A plausible explanation is that the climbers in our sample were more experienced and therefore better able to control their emotional responses to stress.

### Route Preview and Movement Sequence Recall in On-Sight and Red-Point Climbing

In on-sight lead climbing, RI strategy (route preview) before the ascent is an essential skill set to optimize the performance (Boschker et al., 2002; Ferrand et al., 2006; Sanchez et al., 2019). Climbers use route preview to plan the order of climbing movements, determine the best climbing path, improve speed and efficiency, and find rest spots during the route (Boschker et al., 2002). Route finding skills are related to past experiences and motor and perceptive ability. For example, advanced climbers are able to perceive accurately the maximum distance they can reach and program their movements accordingly whereas novices underestimate their reaching capacity (Boschker et al., 2002). In addition, expert climbers are skilled in performing a wider range of technical movements (Sanchez et al., 2019).

Route preview mistakes are one of the major reasons for falling during climbing (Boschker et al., 2002; Sanchez et al., 2012). Indeed, a badly programmed movement during the climb results in: (i) the need for more complex and therefore less economical movements than necessary, (ii) the need to go down to a previous position and to re-set the correct move (with consequent energy expenditure), (iii) a fall, due to excessive complexity of the movement and/or inability to go down. Path strategy planning is linked to strategic effort management (Sanchez et al., 2019).

Previous studies analyzed the influence of route preview only in on-sight climbing (Dupuy and Ripoll, 1989; Sanchez et al., 2012; Seifert et al., 2017). However, when an on-sight climb is unsuccessful, climbers use route preview for the subsequent attempts. It allows the climber to mentally rehearse the move sequences and to reprogram the distribution of effort without physical and psychic expenditure as happens when physically working a route. After the failure of an on-sight climb, the climber can reduce the number of attempts to finish the pitch in red-point mode by optimizing the route preview. This is very important in lead climbing, where the value of performance, both indoor and outdoor, is higher when fewer attempts are needed. It is even more important in bouldering, in both competition and training, in which a key factor in performance is to preserve energy by minimizing the number of attempts on each boulder (White and Olsen, 2010).

In our study, the climbers performed both an on-sight and a red-point ascent preceded and followed by route preview. Analysis of the RI test results indicate that the route-finding skill and the ability to recall movement sequences improve after on-sight (RI_post on–sight_) and before and after red-point ascent (RI_pre red–point_ and RI_post red–point_). We noted a significant decrease in the number of holds incorrectly identified on the route and in the number of holds grasped differently during the climb than previewed (Figure 3). How much these improvements are due to the route preview rather than physical ascent of the pitch is not easy to measure. Nonetheless, the different trend for the two parameters in the RI_pre red–point_ compared to the RI_post on–sight_ suggests that identification of the hand and foot holds and of the best path are easy to retain and to recall, even after time. However, the choice of moves seems more likely to be influenced by short-term motor experience. The technical-physical ability to climb and the interpretative capacity, therefore, may be considered to be trainable and mutually influential aspects.

### Study Limitations

With this experimental protocol, we compared the differences in the on-sight and red-point climb on a route matching the best on-sight skill of each climber. However, we were unable to quantify the influence of the physical experience of climbing from that of RI.

Moreover, we analyzed improvement in RI ability based on an analysis of the dynamic and static phases during ascent and RI test results. We are unable to determine exactly whether the participants used different visual and interpretative strategies. Nevertheless, when we interviewed the participants about their strategies, their accounts agreed with the results of published studies (focus on functional aspects related to movements rather than structural characteristics and spatial information) (Boschker et al., 2002; Pezzulo et al., 2010; Sanchez et al., 2012, 2019; Seifert et al., 2017).

The choice, as participants, of climbers who were both athletes and coaches, may have partially influenced the results. However, the participants having already familiarized themselves with the route preview process and optimization of red-point ascent allowed us to have a uniform sample and to avoid confounding factors.

### Perspectives and Practical Applications

Our findings are shared by recent studies (Sanchez et al., 2012, 2019; Seifert et al., 2017) that reported that route preview ability is a key skill in performance. This is linked also to the new setting trends that draw move sequences in which coordination and motor creativity are increasingly involved, making route preview training methods a priority for coaches and athletes. Furthermore, because optimization of route preview, as mentioned above, has a direct effect on both the economy of individual performances and the overall management of effort during the competitive season, training of this ability will be even more important in view of the new, demanding Olympic combined format.

For this purpose, it could be useful to apply ideo-motor training (Smyth and Waller, 1998; Boschker et al., 2002; Stock and Stock, 2004; Sanchez and Dauby, 2009; Magiera et al., 2013; Sanchez et al., 2019) not only in the usual indoor environment but also in the varying “game context.” For example, climbing in on-sight mode in a natural environment further stimulates the RI ability of the climber, both before and during the ascent, because the movements are not forced by the arrangement and characteristics of the holds set. Even the memory, in the outdoor environment, is further trained, because the wall does not allow easy identification of handholds and footholds as in the climbing-gym. Close to the competition period, in the climbing-gym exercises of RI/movement sequences recall, similar to the tests that we designed for this study, could be proposed. Using visual or technological support, the climber may be asked to retain information, draw paths, propose alternative solutions to the same sequence of holds (as an instructor does with his athletes), or vice-versa imagine different holds sequences that can be resolved with the same motor pattern. Manipulating task and environmental properties, training methods must push the climber out of his comfort zone, to stimulate unusual strategies and solutions to expand his motor and mental patterns.

Previous research (Smyth and Waller, 1998; Boschker et al., 2002; Pezzulo et al., 2010; Seifert et al., 2017) observed that during the route preview, climbers do not focus on the same aspects but rather take advantage of different visual and interpretative strategies. Some try to map the sequencing of holds and determine the spatial objectives useful for climbing, while others focus on weight redistribution and upper and lower limb coordination when planning single economic moves, still others primarily study more complex move sequences. Generally, expert climbers are more focused on the functional aspects of the ascent and novices on the structural characteristics of holds (Boschker et al., 2002).

Overall, differences in climbing skill levels seem to correspond to differences in visual perception and memory. This supports the concept that route preview is a trainable skill and strongly linked to technical-physical abilities. Future evolution of this study could compare the differences in performance and RI ability among climbers of different levels and/or experience.

Furthermore, it could be investigated whether gender influences route preview processes. Several studies showed, both in climbing (Asci et al., 2006) and in other sports (Rice et al., 2019), that female athletes have higher levels of anxiety than males and that this factor affects performance. It is possible, therefore, that higher anxiety in females also affects route preview and interpretative strategies.

## Data Availability Statement

The datasets generated for this study are available on request to the corresponding author.

## Ethics Statement

The studies involving human participants were reviewed and approved by the University of Milan Ethical Committee. The patients/participants provided their written informed consent to participate in this study.

## Author Contributions

EL: conception and design of the study, data acquisition, analysis and interpretation, and drafting of the manuscript. MF: conception and design of the study and data acquisition. SR, EC, SL, and GC: data analysis and interpretation. FE: drafting of the manuscript. All authors reviewed and approved the manuscript.

## Conflict of Interest

The authors declare that the research was conducted in the absence of any commercial or financial relationships that could be construed as a potential conflict of interest.
